# Multiple Precaching Vehicle Selection Scheme Based on Set Ranking in Intermittently Connected Vehicular Networks

**DOI:** 10.3390/s23135800

**Published:** 2023-06-21

**Authors:** Youngju Nam, Jaejeong Bang, Hyunseok Choi, Yongje Shin, Seungmin Oh, Euisin Lee

**Affiliations:** 1 Research Institute for Computer and Information Communication, Chungbuk National University, Cheongju 28644, Republic of Korea; imnyj@chungbuk.ac.kr (Y.N.); hschoi@chungbuk.ac.kr (H.C.); yjshin@chungbuk.ac.kr (Y.S.); 2 Hyundai Autoever, Seoul 06179, Republic of Korea; jaejeongbang@chungbuk.ac.kr; 3 Department of Computer Science and Engineering, Kongju National University, Cheonan 31080, Republic of Korea; smoh@kongju.ac.kr; 4 School of Information Communication Engineering, Chungbuk National University, Cheongju 28644, Republic of Korea

**Keywords:** vehicular ad hoc networks, content precaching, multiple precaching vehicles

## Abstract

In vehicular networks, vehicles download vehicular information for various applications, including safety, convenience, entertainment, and social interaction, from the corresponding content servers via stationary roadside units. Since sufficient RSUs might be difficult to deploy due to rough geographical conditions or high deployment costs, vehicular networks can feature uncovered outage zones between two neighboring RSUs. In these outage zones, vehicles cannot download content, and thus the vehicle networks are defined as intermittently connected vehicular networks. In intermittently connected vehicular networks, the download delay and traffic overhead on the backhaul links are increased due to the large size of the content requested by vehicle users and the long distances between RSUs. Using the mobility information of vehicles, several schemes have been proposed to solve this issue by precaching and relaying content via multiple relaying vehicles in the outage zone. However, because they involved the individual ranking of vehicles for precaching and allocated all of the available precaching amounts to the top-ranking vehicles, they decreased the success rate of content requests and the fairness of vehicle precaching. To overcome the problem of these previous schemes, this paper proposes a multiple precaching vehicle selection (MPVS) scheme that efficiently selects a content-precaching vehicle group with multiple precaching vehicles to precache relayed content in outage zones. To achieve this, we first designed numerical models to decide the necessity and the amount of precaching and to calculate the available precaching amounts of vehicles. Next, MPVS calculates all available vehicle sets and ranks each set based on the available precaching amount. Then, the content-precaching vehicle group is identified from the sets by considering both set rankings and vehicle communication overheads. MPVS also provides a content downloading process through the content-precaching vehicle group in the outage zone. Simulation results conducted in various environments with a content request model and a highway mobility model verified that MPVS was superior to a representative previous scheme.

## 1. Introduction

With the fast development in wireless communications and vehicular technologies, vehicular networks containing smart vehicles such as autonomous cars, equipped with sophisticated sensors and intelligent analysis tools, have evolved to provide intelligent transport services [1,2]. These smart vehicles can drive autonomously on roads with minimal human intervention, ensuring a safer driving experience [3]. As a result, both drivers and passengers can enjoy their travel time without worrying too much about driving, allowing for more leisure time. Through smart vehicles, in-vehicle networks can provide safety and comfort for drivers and passengers and, furthermore, introduce next-generation applications such as multimedia entertainment and social interaction [4]. Generally, vehicular networks consist of stationary roadside units (RSUs) deployed along roads and vehicles moving between them [5]. Vehicles connect to RSUs by vehicle-to-infrastructure (V2I) communications. RSUs function as routers and Internet access points [6]. Through RSUs, vehicles can download interesting content from providers of vehicular applications and services. Recently, the rise in demand for various content in vehicles has led to an increase in mobile data traffic on vehicular networks due to the development of numerous applications and services [7,8,9,10]. This is especially true for larger-sized content with improved quality that is displayed on the larger screens found in smart vehicles. According to the Ericsson Mobility Report, the total traffic on mobile networks is predicted to grow nearly four-fold from approximately 115 exabytes per month at the end of 2022 to 453 exabytes per month by the end of 2028, including fixed wireless access [11]. Of this mobile data traffic, video content is expected to account for about 70 percent, with an estimated increase to 80 percent by 2028.

With the ever-increasing scale of vehicular content, such as multimedia content comprising images, music, and video clips, the communication capacities of vehicular networks might not be able to deliver content to vehicles from a single RSU [12,13]. Vehicular networks must provide seamless connections to vehicles; accordingly, continuous RSUs have been used to overcome this limitation. However, it may be difficult to deploy sufficient RSUs along roads, owing to, e.g., rough geographical conditions or high deployment costs [5]. Vehicular networks presenting these characteristics are defined as intermittently connected vehicular networks (ICVNs) [14,15]. In ICVNs, the distance between the coverage of two neighboring RSUs is defined as the outage zone, and the time that a vehicle spends in this outage zone is termed the outage time [16]. The download delays for vehicles and traffic overhead on the backhaul links are increased due to long-distance outage zones and large content sizes requested by vehicle users. However, as caching capabilities and vehicle mobility prediction improve, ICVNs have an opportunity to address these issues with a content-precaching approach. The content-precaching approach exploits precaching vehicles to download content from an RSU through V2I communications and relay it to a requester vehicle in the outage zone of the RSU coverage by vehicle-to-vehicle (V2V) communications [14].

Many precaching schemes based on the mobility information of vehicles have been proposed for selecting effective precaching vehicles in ICVNs [14,17,18,19,20]. The authors of [17] proposed a scheme named ACSF for selecting a precaching vehicle moving in the same direction as the requester vehicle to achieve minimum outage time. In ACSF, the requester vehicle needed to control its speed in relation to the precaching vehicle to minimize the outage time. In contrast to ACSF, MobTorrent exploited a precaching vehicle moving in the opposite direction and proposed a scheduling algorithm for transferring the precached content using the positions of the requester vehicle and the precaching vehicle [18]. To overcome the limited precaching amount supported by a single precaching vehicle, the authors of [20] proposed a scheme for selecting multiple precaching vehicles moving in the same direction, aiming to provide the maximum amount of the total requested content. The authors of [19] proposed a scheme for using multiple precaching vehicles moving in opposite directions and analyzed its benefits compared to a scheme without precaching. The cooperative store–carry–forward scheme exploited precaching vehicles moving in both the same and opposite directions [14]. However, the existing schemes that select a vehicle group for precaching the requested content allow the selected vehicles to spend all their available resources in supporting other requester vehicles. This results in a high failure rate for the content-precaching requests, because they reduce the resources available to vehicles, causing an imbalance in available resources among the vehicles. In addition, the existing schemes using precaching vehicles moving in the opposite direction increase the failure rate of content delivery, owing to inaccuracies in mobility predictions and connection times.

Therefore, this paper proposes a multiple precaching vehicle selection scheme named MPVS for efficiently selecting a content-precaching vehicle group with multiple precaching vehicles for precaching and relaying content in outage zones of ICVNs, through which high precaching fairness and request success rates can be achieved. To achieve this, we first designed a numerical model to decide the necessity and amount of precaching by comparing the total amount of requested content and the download capacity of the requester vehicle. Next, in the case of content precaching, we determined the available precaching amount of each vehicle in the communication coverage of an RSU, which was used to select the content-precaching vehicle group. Then, the available precaching amount was calculated based on the duration and amount of downloading and relaying according to the mobility information of the vehicle. To select the content-precaching vehicle group with multiple precaching vehicles, in contrast to the existing scheme based on ranking individual vehicles, we next calculated all available sets from the vehicles in the communication coverage of the RSU and ranked each of them based on the total available precaching amount. Then, the content-precaching vehicle group was identified from the sets by considering both the rankings of the sets and the connection overheads with multiple precaching vehicles. With the selected content-precaching vehicle group, we next developed a content downloading process for the requester vehicle in the outage zone. Lastly, we conducted simulations in various environments to evaluate the performance of MPVS. For our simulations, we designed a content request model based on Poisson distribution, Zipf’s law, and Gaussian distribution. Each vehicle decided on a request for its intended content based on Zipf’s law, which reflected its popularity, at any time according to Poisson distribution; the size of the content was dependent on Gaussian distribution. We additionally designed a highway mobility model wherein vehicles traveled on a straight road with acceleration and speed determined based on Gaussian distribution to reflect a realistic highway scenario. By comparing MPVS and adaptive multiple-relay selection (AMRS) [20] based on ranking individual vehicles when selecting multiple precaching vehicles, we verified the superiority of MPVS. The simulation results showed that MPVS achieved better performance than AMRS in terms of precaching fairness and request success rate.

The remainder of the paper is organized as follows: Section 2 reviews the related works on content precaching in content-centric vehicular networks. In Section 3, we present the network model and an overview of our scheme, MPVS. Then, Section 4 describes the four phases of MPVS in detail. To validate the performance of MPVS, Section 5 evaluates the results of the simulation conducted through various environments. Finally, Section 6 concludes the paper.

## 2. Related Work

In vehicular networks, a delay is a very critical issue, because it is directly connected to user safety and the quality of service (QoS) for users. In the case of base-station-centric networks, due to the packet loss and scheduling resulting from an increased demand for larger-sized content, which is hard for a base station (BS) with wireless resource limitations and a wide communication coverage area to meet, vehicles can suffer long delays and buffering, such as in crowded airports and at concerts. When wireless resources are abundant, the huge demand for content can be distributively solved by covering the entire network area through the deployment of many RSUs with a relatively narrow communication coverage area. However, in traditional IP-based networks, vehicles suffer repeated delays in accessing the content server when frequently transitioning between the coverage areas of each RSU due to their high speed of travel.

### 2.1. Content-Centric Vehicular Networks

To address this issue, many researchers have studied CCVNs, which apply the concept of content-centric networks (CCNs [21,22]) to vehicular networks [12,23,24]. By focusing on the content itself instead of the location of the content based on the IP, CCVNs can reduce the delays caused by existing IP-based networks. In CCNs, all nodes have a storage device to cache content in the process of forwarding or providing it according to their scheme. Thus, in CCVNs applying this concept, all RSUs are equipped with a storage device, and all vehicles include storage devices in their onboard units (OBUs). Amadeo et al. [23] hypothesized that adopting a CCN-like strategy could surpass the conventional TCP/IP protocol suite and effectively handle the ever-changing, brief, and sporadic connections encountered in vehicular settings. As a result, a thorough simulation study was conducted to assess the performance of the proposed content-centric vehicular networking architecture. This evaluation encompassed a range of traffic loads, vehicle densities, and content popularity scenarios, aiming to gauge both the effectiveness and efficiency of the approach. Su et al. [12] introduced an innovative framework for a CCVN, unveiling an integrated algorithm for delivering content to vehicles through content-centric units. These units enabled content storage based on priorities determined by vehicle density and content popularity. With the incorporation of a content-centric unit in their CCVN, the management of the content exchanged between vehicles was based on its naming information. Moreover, pending interests were regularly updated by analyzing transmission ratios and network topology. Wang et al. [24] introduced an IP-based framework for vehicular content-centric networking that prioritized the acquisition of content based on a specific position. The framework enabled a requester to acquire content in an address-centric unicast manner, ensuring that the content could be returned to the requester without relying on reverse paths. Additionally, the framework facilitated the retrieval of content from the closest provider at a given position, effectively reducing the cost associated with content acquisition. However, even if the provided and forwarded content was cached, not all content could be cached due to the storage capacity limitation of the RSU. Therefore, the content requested by the vehicle that entered into an RSU for the first time had to be brought from the provider if the content had not been cached in this RSU’s storage; the provider could be the content server or another RSU that possessed the content. The access to the provider was increased because of the frequent handovers due to the high speed of the vehicle, causing access delays and a degraded QoS.

### 2.2. V2I Precaching in CCVNs

In order to reduce this access delay in CCVNs, precaching schemes in RSUs to provide the requested content via V2I communication have been studied by many researchers [2,25,26,27,28,29]. A precaching scheme is a technique that involves proactively caching the content that will be requested by new vehicles before they enter the coverage area. In the context of CCVNs, a precaching scheme can be used to reduce the access delay for the provider when the vehicle enters the coverage area and to improve the reliability of content delivery to the requester vehicle. There have been several approaches to precaching content: (1) the prediction of the request based on the popularity of the content; and (2) the prediction of the next location of the requester vehicle based on its mobility.

#### 2.2.1. Popularity-Based V2I Precaching in CCVNs

First, precaching schemes based on request prediction were studied, aiming to precache content that had a high probability of being requested before the vehicles made the requests [25,26,27]. Ostrovskaya et al. [25] introduced a novel multi-metric content replacement policy (M2CRP) intended for content stores in named data networking-driven VANETs. M2CRP took into account three key metrics that collectively addressed the need for enhanced performance in VANET applications. These metrics included the freshness of the content, its popularity, and the distance between the locations where the content was received and stored in content stores, as well as the current location of the caching node. Amadeo et al. [26] introduced a unique caching strategy called diversity-improved caching of popular transient content (DANTE), which empowered vehicles to independently determine the content to be locally cached based on factors such as content residual lifetime, popularity, and the perceived availability of the same content in the vicinity. They also devised a series of minor modifications in the architecture of named data networking (NDN) nodes and packet fields to facilitate DANTE operations. The caching decisions were made autonomously by the vehicles, enabling them to discover the majority of fresh and popular distinct content nearby without overwhelming the network with content requests that had to reach the original source. Dua et al. [27] introduced a content caching scheme that utilized a bloom filter model, a probabilistic data structure, to enhance the efficiency of content distribution in CCVNs. The bloom filter model was employed to optimize time complexity and accelerate content insertion, deletion, and search operations. By leveraging the bloom filter model, the scheme enabled vehicles to function as caches, facilitating cooperative content distribution among them.

#### 2.2.2. Mobility Prediction-Based V2I Precaching in CCVNs

Mobility prediction-based precaching schemes have been investigated to precache the content that will be requested by predicting the movement of vehicles [2,28,29]. Zhe et al. [2] presented an innovative hierarchical proactive caching approach that took into account both the mobility patterns and future demands of autonomous vehicle users. This approach employed non-negative matrix factorization (NMF) to predict users’ preferences, which were then utilized to anticipate their future demands considering the historical popularity of videos. By considering the user’s current velocity vector, the approach calculated the number of video chunks for precaching based on the user’s arrival and departure times at an edge node. In addition to predicted ratings, the approach incorporated the past popularity of videos to enhance the accuracy of predicting users’ future demands. Park et al. [28] introduced a mobility-aware distributed proactive caching scheme for CCVNs. The proposed scheme aimed to reduce redundancy and minimize the precaching burden on multiple candidate upcoming RSUs following the current RSU. To achieve this, the scheme distributed the intended content proportionally to each candidate RSU based on the mobility probability of the requester vehicle. This probability represented the likelihood of the requester vehicle transitioning from the current RSU to the candidate RSU, as determined by a Markov model. By considering the constant speed of the vehicles, the scheme calculated the maximum number of content chunks. These chunks were then distributively precached across the candidate RSUs that the requester vehicle could potentially visit next. Khelifi et al. [29] proposed an optimized precaching scheme called PCMP, designed for VANETs within the NDN architecture. This scheme utilized a long short-term memory (LSTM) module to predict the next arrival RSU and subsequently precached the intended content in that RSU. To determine the number of chunks, the scheme took into account the current velocity of the requester vehicle and the distance of the path within the coverage of the RSU. PCMP divided the intended content into chunks and calculated the precise number of chunks that should be both precached and downloaded at the next RSU. This calculation was based on factors such as the connection time of the requester vehicle within the RSU’s coverage area and the link bandwidth between the vehicle and the RSU. However, these existing precaching schemes have limitations, because the entire network area cannot be covered by RSUs due to the cost of RSU deployment. An area that is not covered by RSUs is called an outage zone or a dark area. In these areas, the requester vehicle must consume costly wireless resources from a BS or suffer a long delay by not receiving the requested content.

### 2.3. V2V Precaching Using One Relaying Vehicle in CCVNs

To cover these outage zones, many researchers have studied precaching schemes involving other vehicles to provide the requested content via V2V communication within outage zones in CCVNs [16,17,19,20,30]. The vehicle selected to provide the content by precaching it is called the relaying vehicle. First, several researchers studied V2V precaching schemes, which select one vehicle to provide the requested content to the requester vehicle by precaching it [16,17,30]. Wu et al. [17] presented an adaptive carry–store–forward scheme that selected the vehicle that remained in an RSU’s range the longest as the relaying vehicle. The RSU waited until the requester vehicle was in the middle of the RSU’s coverage area and then selected a relaying vehicle that would remain in this area the longest among the vehicles behind the requester vehicle. It then changed the speed of the requester vehicle to match the speed of the selected relaying vehicle in order to maximize the coverage of the outage zone. However, it is not practical to change the speed of a vehicle for content provision reasons. Bang et al. [16] proposed that the vehicle selected as the relaying vehicle should be the one that could deliver the largest amount of the requested content to the requesting vehicle. Their scheme compared the amount of content a candidate vehicle could precache until the requester vehicle was out of the range of the current RSU when the requester vehicle requested the content and the amount that could be delivered based on the connection time between the candidate vehicle and the requester vehicle. Therefore, the vehicle that could deliver the most content was selected as the relaying vehicle to cover as much of the outage zone as possible. Nam et al. [30] proposed a solution to additionally precache the requested content by considering the mobility error of the relaying vehicle and the requester vehicle. In this scheme, the relaying vehicle was selected as the vehicle that could provide the most precached content to the requester vehicle in the same way as in the previous paper. In order to avoid incurring overhead by performing too much additional precaching, the scheme tried to cover as much of the outage zone as possible by precaching the content to be delivered in the extended connection time caused by the mobility error of the vehicles with an appropriate amount of additional precaching.

### 2.4. V2V Precaching Using Relaying Vehicles in CCVNs

To improve the performance in covering outage zones, many researchers have studied V2V precaching schemes, which select multiple vehicles as relaying vehicles [14,19,20]. Guo et al. [19] exploited the combination of precaching and carry-and-forward schemes to facilitate data downloading by an individual vehicle in dark areas. When a requester vehicle requested to download data, it first informed multiple selected RSUs to precache the data; then, relaying vehicles were selected to form linear clusters and cooperatively download the precached data from the selected RSUs. When the requester vehicle left the coverage of an RSU, it continued to download data from cooperative clusters encountered in the dark area, which indirectly extended the access time between the requester vehicle and the RSUs, accordingly minimizing the dark areas. Wang et al. [14] suggested selecting two relaying vehicles, one traveling in the same direction as the requester vehicle and another coming from the opposite direction. To avoid overlaps in the periods during which the two vehicles delivered content to the requester vehicle, the scheme calculated the delivery capacity of the vehicle coming from the opposite direction from the time at which the vehicle traveling in the same direction finished delivering content. The outage zone was reduced by considering one additional vehicle coming from the opposite direction. Ahmed et al. [20] proposed a scheme to cover outage zones using vehicles traveling in the same direction as the relaying vehicle as much as possible. The scheme calculated the amount of content that could be delivered from each candidate vehicle to the requester vehicle and used the vehicles with the highest capacity as relaying vehicles. Multiple vehicles were selected to cover the amount of content that needed to be delivered in the outage zone. However, existing V2V precaching schemes ignore the fact that the vehicles selected as relaying vehicles can also intend to request content. According to these schemes, relaying vehicles spend all of their resources while traveling within the RSU’s coverage area precaching the content requested by other vehicles. A relaying vehicle that spends all of its resources in this way cannot request its own content. Therefore, the vehicle suffers a very long delay, because it has to request its content within the coverage area of the next RSU.

### 2.5. Contributions

In this paper, we introduce the MPVS scheme to guarantee high fairness for every vehicle. To achieve this purpose, our contributions are as follows:

- We considered all groups that could become a content-precaching vehicle group based on their ability to provide the requested content within the outage zone. We prioritized selecting groups containing fewer relaying vehicles to ensure a high success rate for content requests and improve the overall performance.
- We set the proportion of dwell time used to precache the requested content by each relaying vehicle in the selected group within the current RSU’s coverage. This was in contrast to existing V2V precaching schemes, wherein all the abundant dwell time of relaying vehicles was spent precaching, resulting in an inability to request or receive their own content. By controlling the proportion of dwell time used by each relaying vehicle, we improved the fairness of content request allocation.
- We designed a content request model to evaluate the proposed scheme. Each vehicle decided whether to request content at a given time according to Poisson distribution. When the vehicle decided to request content, the requested content was decided based on Zipf’s law. Then, the size of the content was decided based on Gaussian distribution.
- We designed a highway mobility model wherein vehicles traveled with acceleration and speed determined based on Gaussian distribution, reflecting a realistic highway scenario. We also considered a highway scenario that had no drastic directional changes, with all vehicles traveling on a straight road.

## 3. Network Model and Scheme Overview

In this section, we present the network model and overview of the proposed scheme, MPVS.

### 3.1. Network Model

**Figure 1:**
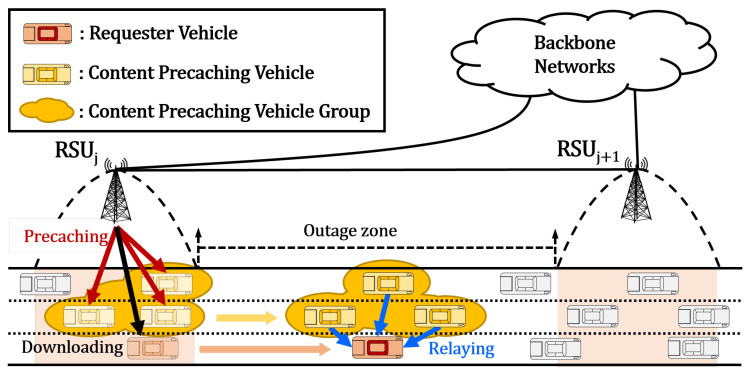
Network model of the proposed MPVS scheme: The black arrow denotes that the requester vehicle downloads the intended content from the current RSU; The red arrow denotes that the content precaching vehicles precache the requested content for the requester vehicle; The blue arrow denotes that the content precaching vehicles forward the precached content to the requester vehicle.

In this paper, we considered the network shown in Figure 1 as the network model for the proposed CCVN scheme. This network model consisted of a large number of vehicles moving on roads and many RSUs located beside the roads [6,31]. Vehicles traveled along their own trajectories to their own destinations by passing several RSUs. All vehicles had different speeds and changed their speeds according to the traffic situations on the roads. To achieve this, the speeds of the vehicles on a road were divided into *L* discrete levels within a range from 0 km/h to the maximum regulation speed of the road. We defined the set of vehicle speed levels as $S_{L}=\left\{1,2,3,\ldots ,L\right\}$. A vehicle on this road randomly selected a speed level from $S_{L}$ and moved with the selected speed level on the road. RSUs were deployed along the roads, and each pair of neighboring RSUs had a regular distance between them [32]. Every RSU had a communication range to cover a specific distance along the roads. Vehicles could communicate with each other through V2V wireless communication and communicate with RSUs through V2I wireless communication. RSUs could communicate with each other and content servers through wired communication provided by backhaul links and could communicate with vehicles through infrastructure-to-vehicle (I2V) wireless communication. In our scheme, every vehicle sent a beacon message with its ID, location, and mobility information to its neighboring vehicles periodically. Thus, every vehicle could be aware of its neighboring vehicles and save the information about them extracted from the beacon messages in its neighbor table. Every RSU sent a solicitation message to vehicles in its communication coverage periodically. Thus, when a vehicle entered the communication coverage of an RSU, it received a solicitation message from the RSU. On receiving the solicitation message, the vehicle sent a beacon message with its ID, location, and mobility information to the RSU. Through this process, an RSU could also be aware of the vehicles in its communication coverage and save the information about them extracted from the beacon messages in its neighbor table.

Generally, RSUs cannot be deployed across the whole area covered by roads in CCVNs owing to several geographical constraints and high deployment costs. As a result, two neighboring RSUs might have an outage zone between them, as shown in Figure 1, which lies outside the communication coverage areas of both [16]. In an outage zone, vehicles cannot be covered by any of the RSUs for I2V and V2I communication. If a vehicle wants to download requested content, when it enters the communication coverage area of an RSU, it can request the content from the RSU. On receiving the content download request, the RSU retrieves the content from the content server and downloads it to the vehicle through I2V communication. When the intended content is large, the RSU cannot fully download it to the requester vehicle due to the limited transmission rate of I2V communication. In this case, after the requester vehicle leaves the communication coverage area of the RSU, it can no longer download the content because it is traveling in the outage zone between two RSUs. When the vehicle enters the communication coverage area of the next RSU, it can continue downloading the content from this RSU. Thus, the traveling time in the outage zones represents the delay in downloading the content. If the distance of the outage zones increases, the delay also increases. To reduce the delay in the outage zones, MPVS selects a content-precaching vehicle group comprising multiple vehicles in the communication coverage area of an RSU and precaches the intended content for the requester vehicle. In the outage zone, each vehicle in the content-precaching vehicle group relays the precached content to the requester vehicle. As a result, the delay in downloading the content in the outage zone can be reduced by the content-precaching vehicle group. For MPVS, we assumed that all vehicles and RSUs used IEEE 802.11p wireless access in vehicular environments (WAVE) communication [33]. The downloading transmission rate of an RSU was the same for every vehicle in its communication coverage area. Generally, the communication range of RSUs is wider than that of vehicles, because the transmission power and rate of RSUs are higher than those of vehicles. In other words, I2V communication coverage is greater than V2V communication coverage.

### 3.2. Scheme Overview

If a vehicle wants to download its intended content while moving to its destination, it makes an interest packet for the intended content. When it enters the communication coverage area of an RSU, it sends the interest packet to the RSU to request the intended content. In this paper, we refer to a vehicle sending an interest packet to an RSU as a requester vehicle. On receiving the interest packet, the RSU checks whether it has the content in its caching storage. If the RSU does not have the content, it also requests, downloads, and saves the content from a corresponding content server or another RSU that has the content. Once it possesses the content, the RSU checks whether it has enough time to provide all of the requested content to the requester vehicle and decides the necessity of precaching the content at the next RSU. To achieve this, it judges whether it can download all of the content to the requester vehicle in its communication coverage area by comparing the total amount of requested content and the amount downloadable by the requester vehicle in its communication coverage area. The downloadable amount for the requester vehicle is calculated using both the remaining travel time in the communication coverage area of the RSU and the V2I transmission rate of the RSU. If the downloadable amount is larger than the total amount, then the RSU can fully download all of the content to the requester vehicle in its communication coverage area and does not need to select a content-precaching vehicle group. Thus, the requester vehicle can finish the content downloading process in the communication coverage area of the RSU.

On the other hand, if the total amount is larger than the downloadable amount, then the RSU cannot fully download all of the content to the requester vehicle in its communication coverage area and needs to select a content-precaching vehicle group to relay the precaching content to the requester vehicle in the outage zone. When the RSU selects the content-precaching vehicle group for the content requested by the requester vehicle, it ranks all possible sets formed by all the vehicles in its communication coverage area. The ranking of a set is determined by the total available precaching amounts of the vehicles in the set. An appropriate content-precaching vehicle group is selected from all sets by considering both the set rankings determined by the content-precaching amounts of the vehicles and the communication overheads caused by connections with multiple vehicles in order to achieve the performance goals of MPVS. Then, the RSU fairly allocates the precaching amounts for each vehicle in the set selected as the content-precaching vehicle group in proportion to the vehicle’s available precaching amount. As shown in Figure 1, every vehicle in the content-precaching vehicle group relays its precaching amount to the requester vehicle in the outage zone when the two vehicles can communicate with each other. In this situation, the requester vehicle can finish the content downloading process in the outage zone. However, even if the content-precaching vehicle group with all its vehicles in the communication coverage area of the RSU cannot download all of the content to the requester vehicle in the outage zone, the RSU can precache the remaining amount (i.e., discounting the amount downloaded by the RSU and the amount relayed by the content-precaching vehicle group) to the next RSU through backhaul links. When the requester vehicle enters the communication coverage area of the next RSU, the next RSU conducts the content downloading process with the remaining amount, as in the previous RSU. This process continues until the requester vehicle downloads the total amount of requested content. We describe the proposed MPVS scheme in detail in Section 4.

## 4. MPVS Scheme

In this section, we describe the MPVS scheme. MPVS consists of four phases: (1) the calculation of the downloadable content amount, (2) the calculation of the content-precaching amount, (3) the selection of a content-precaching vehicle group, and (4) the downloading of content through the content-precaching vehicle group. The first phase is to calculate the amount of content that a vehicle can download while within the RSU’s coverage area, in order to determine the necessity of selecting a content-precaching vehicle group. The second phase is to calculate the content-precaching amount of every vehicle in the communication coverage area of the RSU, which is needed to select a content-precaching vehicle group. The third phase is to select the content-precaching vehicle group based on both the set rankings determined by the content-precaching amounts of the vehicles and the communication overheads caused by connections with multiple vehicles. Lastly, the fourth phase is to provide the downloaded content to the requester vehicle through the content-precaching vehicle group in the outage zone. We sequentially present the four phases in detail in the following four subsections. Table 1 shows the notation used for MPVS.

### 4.1. Calculation of Content Downloading Amount

Generally, a requester vehicle $V_{req}$ wants to download the total amount $TA_{C_{c}}$ of the intended content $C_{c}$ while moving along its trajectory toward its destination. To download the content, $V_{req}$ sends a request packet with information on its ID, its position, its mobility, and the content’s ID and size to the RSU to which it currently belongs ($RSU_{j}$). On receiving the request packet, $RSU_{j}$ checks whether it needs to select a content-precaching vehicle group for $V_{req}$ based on the information contained in the request packet. To achieve this, it first calculates the remaining time $RT_{req}$ for which $V_{req}$ is expected to be located within the coverage of $RSU_{j}$ as follows:

(1) $$RT_{req}=\frac{x_{EX_{j}}-x_{req}}{v_{req}},$$

where $x_{EX_{j}}$ is the end point of the coverage area of $RSU_{j}$ in the moving direction of $V_{req}$, $x_{req}$ is the position of $V_{req}$, and $v_{req}$ is the speed of $V_{req}$.

Next, $RSU_{j}$ calculates the amount $DA_{req}$ that $V_{req}$ can download from $RSU_{j}$ within its communication coverage area during the remaining time $RT_{req}$ as follows:

(2) $$DA_{req}=RT_{req}\times R_{I2V},$$

where $R_{I2V}$ is the transmission rate of infrastructure-to-vehicle (I2V) communication in ICVNs. Following existing works [20], we assumed that $R_{I2V}$ is constant regardless of the distance between an RSU and a vehicle within the communication coverage area of the RSU.

After obtaining the downloadable amount $DA_{req}$ for $V_{req}$, $RSU_{j}$ determines whether it needs to select a content-precaching vehicle group in order to enable the total content amount $TA_{C_{c}}$ to be completely downloaded to $V_{req}$. To achieve this, $RSU_{j}$ compares $DA_{req}$ with $TA_{C_{c}}$. If $TA_{C_{c}}$ ≤ $DA_{req}$, then $RSU_{j}$ can completely deliver the total size $TA_{C_{c}}$ of the content $C_{c}$ to $V_{req}$ through the $R_{I2V}$ communication in its communication coverage area. In this case, $RSU_{j}$ downloads the total amount $TA_{C_{c}}$ of the content $C_{c}$ to $V_{req}$ while $V_{req}$ is traveling within its communication coverage area. When it has fully received the content $C_{c}$, $V_{req}$ can immediately exploit it. However, if $TA_{C_{c}}$ > $DA_{req}$, then $RSU_{j}$ cannot fully deliver $TA_{C_{c}}$ to $V_{req}$ in its communication coverage area. Thus, to complete the downloading of $TA_{C_{c}}$ to $V_{req}$, $RSU_{j}$ selects a content-precaching vehicle group consisting of multiple vehicles located in the coverage area of $RSU_{j}$ and sends the remaining amount $\left(RMA_{C_{c}}=TA_{C_{c}}-DA_{req}\right)$ of the content to the selected precaching vehicle group. On receiving the remaining content amount $RMA_{C_{c}}$, each vehicle in the content-precaching vehicle group relays the rest of the content to $V_{req}$ outside the $R_{I2V}$ communication coverage area (i.e., in the outage zone) after $V_{req}$ exits the coverage area of $RSU_{j}$. In Section 4.2, we present a method to calculate the available precaching amount of vehicles needed to select a content-precaching vehicle group in MPVS. Then, we present a method to select an optimal content-precaching vehicle group based on the available precaching amount in Section 4.3.

### 4.2. Calculation of Content-Precaching Amount

To deliver the remaining amount $RMA_{C_{c}}$ of the content $C_{c}$ to $V_{req}$, $RSU_{j}$ selects a content-precaching vehicle group from all of the vehicles in its coverage area. In contrast to MPVS, the existing scheme AMRS [20] ranked each vehicle individually according to its available storage size and selected multiple vehicles for the precaching vehicle group based on the individual vehicle rankings to deliver as much of the $RMA_{C_{c}}$ as possible to $V_{req}$. However, in AMRS, the multiple selected precaching vehicles consumed almost all of their available storage precaching $RMA_{C_{c}}$. As a result, they could not use their own storage to save their own content and, furthermore, they could not be exploited to precache content for other requester vehicles. Conversely, MPVS selects a content-precaching vehicle group based on the ranking of sets comprising multiple vehicles, rather than the ranking of individual vehicles. MPVS forms all possible sets of vehicles, ranks each set according to the criteria to achieve its precaching purpose, and selects a content-precaching vehicle group based on the ranking of the sets. As the criteria for ranking sets, MPVS uses the available precaching amount of each vehicle, which is calculated based on the amount of content that can be downloaded and relayed by the vehicles.

To determine the available precaching amount of a vehicle $V_{i}\left(i=1,2,\cdots ,n\right)$ in the communication coverage area of $RSU_{j}$, where $V_{req}$ is located, one first calculates the downloading time and downloadable amount of $V_{i}$. The downloading time $DT_{i}$ of $V_{i}$ is defined as the time for which $V_{i}$ can download content from $RSU_{j}$ before it passes the exit point $x_{EX_{j}}$ of $RSU_{j}$ in its moving direction. $RSU_{j}$ calculates the downloading time $DT_{i}$ for $V_{i}$ based on the position and speed of $V_{i}$ as follows:

(3) $$DT_{i}=\frac{x_{EX_{j}}-x_{i}}{v_{i}},$$

where $x_{i}$ is the position of $V_{i}$, and $v_{i}$ is the speed of $V_{i}$. Then, $RSU_{j}$ calculates the downloadable amount $DA_{i}$ of content that $RSU_{j}$ can provide to $V_{i}$ using $DT_{i}$ and $R_{I2V}$, as follows:

(4) $$DA_{i}=DT_{i}\times R_{I2V}.$$

Since every vehicle might have a different storage capacity, $V_{i}$ has its own storage capacity $CS_{i}$. Accordingly, the practical downloadable amount of content for $V_{i}$ is selected as the smaller value between $DA_{i}$ and $CS_{i}$, as follows:

(5) $$PDA_{i}=min\left\{DA_{i},CS_{i}\right\},\left(i=1,2,\cdots ,n\right).$$

Thus, $C_{ADV_{i}}$ is the amount of content that $V_{i}$ can actually download from $RSU_{j}$.

Next, one calculates the relaying time and amount of a vehicle $V_{i}\left(i=1,2,\cdots ,n\right)$ to the requester vehicle $V_{req}$ in the outage zone after $V_{req}$ leaves the coverage of $RSU_{j}$. The relaying time $RT_{i}$ of $V_{i}$ is defined as the duration for which $V_{i}$ can relay the precached content to $V_{req}$ in the outage zone. $RSU_{j}$ calculates $RT_{i}$ based on the position and speed information of both $V_{req}$ and $V_{i}$ as follows:

(6) $$RT_{i}=\frac{r_{c}-\left|x_{req}-x_{i}\right|}{\left|v_{req}-v_{i}\right|},$$

where $r_{c}$ is the communication range of the vehicles. However, $RT_{i}$ can sometimes be longer than the time for which $V_{req}$ can stay in the outage zone, because $V_{i}$ and $V_{req}$ can maintain a continuous connection due to their similar speeds. In this case, $RT_{i}$ is set to $t_{max}$, which is calculated as follows:

(7) $$t_{max}=\frac{U}{v_{req}}-\left|DT_{req}-DT_{i}\right|,$$

where *U* is the distance of the outage zone between $RSU_{j}$ and the next RSU, $RSU_{j+1}$. In Equation (7), $\frac{U}{v_{req}}$ is the time that $V_{req}$ consumes to pass *U*. After both $v_{req}$ and $v_{i}$ leave the coverage area of $RSU_{j}$, $v_{i}$ can relay the precached content to $v_{req}$. In Equation (7), $|DT_{req}-DT_{i}|$ is the time taken for both $v_{req}$ and $v_{i}$ to leave the coverage area of $RSU_{j}$. Since $v_{i}$ can be located infront or behind $v_{req}$, $DT_{req}-DT_{i}$ has an absolute value. Having obtained $RT_{i}$, $RSU_{j}$ then calculates the relaying amount $RA_{i}$, i.e., the content that $V_{i}$ can provide to $V_{req}$ in the outage zone, as follows:

(8) $$RA_{i}=RT_{i}\times R_{V2V},$$

where $R_{V2V}$ is the transmission rate of V2V communication.

Using Equations (5) and (8), $RSU_{j}$ obtains information about both the downloadable amount $PDA_{i}$ and the relaying amount $RA_{i}$ of each vehicle $V_{i}$ in its coverage area as follows:

(9) $$V_{i}=\left\{PDA_{i},RA_{i}\right\},\left(i=1,2,\cdots ,n\right).$$

Practically, $V_{i}$ can provide only the smaller amount between $PDA_{i}$ and $RA_{i}$ to $V_{req}$ in the outage zone. Thus, the precaching amount $PA_{i}$ of each $V_{i}$ in the coverage area of $RSU_{j}$ is decided as follows:

(10) $$PA_{i}=min\left\{PDA_{i},RA_{i}\right\},\left(i=1,2,\cdots ,n\right).$$

In MPVS, $RSU_{j}$ selects a content-precaching vehicle group based on the precaching amount $PA_{i}$ of each vehicle $V_{i}$ for $V_{req}$ in its communication coverage area. We explain the method for selecting a content-precaching vehicle group in the next subsection.

### 4.3. Selection of Content-Precaching Vehicle Group

In MPVS, when $RSU_{j}$ determines a content-precaching vehicle group for a requester vehicle $V_{req}$, it exploits the ranking of sets comprising multiple vehicles in its communication coverage area. Based on the ranking of sets, MPVS then selects the content-precaching vehicle group to precache the remaining amount $RMA_{C_{c}}$ of the content $C_{c}$ for $V_{req}$. To achieve this, $RSU_{j}$ first forms a set $S_{V_{j}}$ of every vehicle $V_{i}$ with its precaching amount $PA_{i}$ within the coverage area as follows:

(11) $$S_{V_{j}}=\left\{PA_{i}|i\in \left[1,N\right]\right\}.$$

Then, $RSU_{j}$ considers a set $R_{j}$ of all available groups formed by $S_{V_{j}}$ for precaching as follows:

(12) $$R_{j}=\left\{r\left(p,q\right)|p\in \left[1,N\right],q\in \left[1{,}_{N}C_{p}\right]\right\},$$

where *N* is the maximum value of *p* and can be denoted as $N=|S_{V_{j}}|$; $R_{j}$ is the power set of $S_{V_{j}}$; *p* and *q* are the number of vehicles in the subset and the order of groups with the same number of vehicles, respectively; r(p,q) is an element of the power set; and $|S_{V_{j}}|$ is the number of elements in $S_{V_{j}}$. For example, let $RSU_{j}$ contain three vehicles $V_{1}$ with $PA_{1}$, $V_{2}$ with $PA_{2}$, and $V_{3}$ with $PA_{3}$ in its coverage area. Then, $S_{V_{j}}=\left\{PA_{1},PA_{2},PA_{3}\right\}$. Accordingly, all elements of the power set $R_{j}$ are $r\left(1,1\right)=\left\{PA_{1}\right\}$, $r\left(1,2\right)=\left\{PA_{2}\right\}$, $r\left(1,3\right)=\left\{PA_{3}\right\}$, $r\left(2,1\right)=\left\{PA_{1},PA_{2}\right\}$, $r\left(2,2\right)=\left\{PA_{1},PA_{3}\right\}$, $r\left(2,3\right)=\left\{PA_{2},PA_{1}\right\}$, and $r\left(3,1\right)=\left\{PA_{1},PA_{2},PA_{3}\right\}$.

We define these element sets in $R_{j}$ as candidate sets to be selected as a content-precaching vehicle group for $V_{req}$. $RSU_{j}$ selects the most appropriate among them for the content precaching to deliver $RMA_{C_{c}}$ to $V_{req}$ in the outage zone. Generally, every candidate set might have a different precaching amount, because the vehicles in each set have different precaching amounts. As the content-precaching vehicle group, MPVS can choose from all candidate sets those that have content-precaching amounts above $RMA_{C_{c}}$, so that every vehicle in the selected sets can additionally use its precaching amount for itself and other requester vehicles even after consuming its precaching amount for the initial requester vehicle. In other words, $V_{i}$ can allow its $PA_{i}$ to remain as high as possible even after being applied in content precaching for $V_{req}$. Then, to determine the content-precaching vehicle group, $RSU_{j}$ calculates whether the vehicles in each candidate set r(p,q) have sufficient available precaching amounts to precache the remaining amount $RMA_{C_{c}}$, as follows:

(13) $$ss\left(r\left(p,q\right)\right)=\left\{\begin{array}{cc} 1, & \mathrm{if}\;\sum_{n=1}^{K}PA_{n}\geq RMA_{C_{c}},\;K=|r(p,q)|\\ 0, & otherwise, \end{array}\right.$$

where K=∣r(p,q)∣ is the number of elements in the set r(p,q), and ss(r(p,q)) evaluates whether r(p,q) has a sufficient available precaching amount. As shown in Equation (13), if r(p,q) has a sufficient available precaching amount, ss(r(p,q))=1. Otherwise, ss(r(p,q))=0. If any r(p,q) has an insufficient available precaching amount, the total precaching amount of all vehicles in the communication coverage area of $RSU_{j}$ is less than $RMA_{C_{c}}$. Then, $RSU_{j}$ selects all the element vehicles in r(N,1) as the content-precaching vehicle group $L_{CPVG}$ to provide as much of the remaining amount $RMA_{C_{c}}$ of the requested content $C_{c}$ as possible within the outage zone. In other words, $RSU_{j}$ selects all vehicles in its communication coverage area (that is, all the element vehicles in $S_{V_{j}}$) as $L_{CPVG}$. In this case, the vehicles in $L_{CPVG}$ (that is, the set r(N,1)) can precache $RMA_{C_{c}}$ and relay it to $V_{req}$ in the outage zone. Then, the total available precaching amount $TPA_{L_{CPVG}}$ of all vehicles in $L_{CPVG}$ is calculated as follows:

(14) $$TPA_{L_{CPVG}}=\sum_{n=1}^{K}PA_{n}.$$

Each vehicle $V_{i}$ in $L_{CPVG}$ downloads its available precaching amount $PA_{i}$ for $RMA_{C_{c}}$ from $RSU_{j}$ and relays $PA_{i}$ to $V_{req}$ in the outage zone. However, even after precaching $TPA_{L_{CPVG}}$, the remaining amount $RMA_{C_{c}}$ of the requested content $C_{c}$ is yet to be downloaded, calculated as $RMA_{C_{c}}-\sum_{n=1}^{K}PA_{n}$, because $RMA_{C_{c}}>\sum_{n=1}^{K}PA_{n}$. $\left(RMA_{C_{c}}-\sum_{n=1}^{K}PA_{n}\right)$ needs to be precached to the next RSU $RSU_{j+1}$ after $RSU_{j}$ and thus is defined as the RSU precaching amount $RPA_{j+1}$ of $RSU_{j+1}$. Thus, $RSU_{j}$ precaches $RPA_{j+1}$ to $RSU_{j+1}$ through backhaul links. When $V_{req}$ enters the communication coverage area of $RSU_{j+1}$, $RSU_{j+1}$ continues the content downloading process with $RPA_{j+1}$ for $V_{req}$.

On the other hand, if multiple candidate sets r(p,q) have sufficient available precaching amounts (that is, they have ss(r(p,q))=1), $RSU_{j}$ has to select one of them as the content-precaching vehicle group, because they can all provide the total remaining amount $RMA_{C_{c}}$ in the outage zone. To achieve this, $RSU_{j}$ first chooses the candidate sets r(p,q) with ss(r(p,q))=1 and defines a set containing all of them as the sufficient set $SR_{j}$. Accordingly, $SR_{j}$ is defined as follows:

(15) $$SR_{j}=\left\{r\left(p,q\right)|\left(r\left(p,q\right)\in R_{j}\right)\cap \left(ss\left(r\left(p,q\right)\right)=1\right)\right\}.$$

Next, one chooses the sets with the lowest number of vehicles among all sets in $SR_{j}$. If a set with many vehicles is used as the content-precaching vehicle group, $V_{req}$ has to communicate with many vehicles to obtain the precached content, and so the communication process generates a lot of overhead. Thus, $\alpha_{min}$ is defined as the lowest *p* value in r(p,q), i.e., the sets with the lowest number of vehicles in $SR_{j}$, as follows:

(16) $$\alpha_{min}=min\left\{r\left(p,q\right)|r\left(p,q\right)\in SR_{j}\right\}.$$

Then, $RSU_{j}$ chooses the sets with the lowest number (i.e., $\alpha_{min}=p$) of vehicles in $SR_{j}$ and uses them to build set $LR_{j}$.

(17) $$LR_{j}=\left\{r\left(p,q\right)|\left(r\left(p,q\right)\in SR_{j}\right)\cap \left(\alpha_{min}=p\right)\right\}.$$

Next, $RSU_{j}$ calculates the sum of the remaining precaching amounts of all the vehicles in each set of $LR_{j}$ except the remaining amount $RMA_{C_{c}}$ to be delivered to $V_{req}$ in the outage zone, as follows:

(18) $$S\left(r\left(p,q\right)\right)=\sum_{n=1}^{p}\left(PA_{n}-RMA_{C_{c}}\times \frac{PA_{n}}{\sum_{m=1}^{p}PA_{m}}\right),r\left(p,q\right)\in LR_{j}.$$

To fairy allocate a smaller content precaching load to vehicles in the content-precaching vehicle group, the scheme selects the set from all sets in $LR_{j}$ that has the largest total available precaching amount among its element vehicles and thus retains the largest available precaching amount after precaching $RMA_{C_{c}}$. Ultimately, $RSU_{j}$ selects the set with the largest remaining precaching amount among all sets in $LR_{j}$ as the content-precaching vehicle group $L_{CPVG}$ as follows:

(19) $$L_{CPVG}=\left\{r\left(p,q\right)|\arg max_{\forall S(r(p,q))},r\left(p,q\right)\in LR_{j}\right\}.$$

If $L_{CPVG}$ is selected to precache $RMA_{C_{c}}$ for $V_{req}$, $RSU_{j}$ fairly allocates the precaching amount $\left(RMA_{C_{c}}\times PA_{i}/\sum_{m=1}^{p}PA_{m}\right)$ as the precaching amount for each vehicle $V_{i}$ in $L_{CPVG}$ in proportion to its available precaching amount $PA_{i}$ for $RMA_{C_{c}}$. Eventually, $RSU_{j}$ sends the $\left(RMA_{C_{c}}\times PA_{i}/\sum_{m=1}^{p}PA_{m}\right)$ of $RMA_{C_{c}}$ to each vehicle $V_{i}$ in $L_{CPVG}$.

For example, if a requester vehicle requests 150 MB of content and there are three candidate vehicles that could deliver the content within the outage zone, assuming that the three candidate vehicles have $PA_{1}=50$, $PA_{2}=90$, and $PA_{3}=100$, respectively, then $S_{V_{j}}=50,90,100$. Accordingly, all elements of the power set $R_{j}$ are r(1,1)=50, r(1,2)=90, r(1,3)=100, r(2,1)=50,90, r(2,2)=50,100, r(2,3)=90,100, and r(3,1)=50,90,100. ss(r(p,q)) is decided as follows: ss(r(1,1))=0, ss(r(1,2))=0, ss(r(1,3))=0, ss(r(2,1))=0, ss(r(2,2))=1, ss(r(2,3))=1, and ss(r(3,1))=1, because the requested content size is 150 MB. Therefore, only sets that have ss(r(2,2))=1, ss(r(2,3))=1, and ss(r(3,1))=1 are considered for the content-precaching vehicle group. Among the three sets, the scheme selects the sets with the lowest number of vehicles, denoted as r(2,2) and r(2,3). Between these two sets, the set that has the largest available precaching amount is selected as the content-precaching vehicle group, which is r(2,3). In the existing schemes [20], $V_{3}$ with $PA_{3}=100$ would spend all of its dwell time, leading to request failure. To guarantee fairness, we set $RA_{C_{2}}$ as 150×(90/190)=71.05 and $RA_{C_{3}}$ as 150×(100/190)=78.95, that is, 78.95 % of each $PA_{i}$, and they could use the remaining proportion of their dwell time for requesting their own content. $V_{2}$ relays 71.05 MB of the requested content to $V_{req}$ within the outage zone, and $V_{3}$ relays 78.95 MB of the requested content to $V_{req}$ within the outage zone. In the following subsection, we explain the method through which the precaching amounts are relayed to $V_{req}$ from the vehicles in $L_{CPVG}$ in the outage area so that it can download $RMA_{C_{c}}$.

### 4.4. Content Downloading through Content-Precaching Vehicle Group

Usually, a requester vehicle $V_{req}$ can request its intended content $C_{c}$ when it enters the communication coverage area of an RSU $RSU_{j}$. To make the request, $V_{req}$ sends a request packet with information about both itself (i.e., ID, location, destination, mobility, etc.) and $C_{c}$ (i.e., ID, size, etc.) to $RSU_{j}$. On receiving the request packet, $RSU_{j}$ sends an ACK packet to $V_{req}$ in response to the request. Then, to start the process of downloading $C_{c}$ to $V_{req}$, $RSU_{j}$ first checks whether it has $C_{c}$ in its caching storage. If it does not have $C_{c}$, it downloads $C_{c}$ from a content server or another RSU that has $C_{c}$ and saves $C_{c}$ in its caching storage. Next, $RSU_{j}$ calculates the amount $DA_{req}$ that $V_{req}$ can download in the communication coverage area of $RSU_{j}$. Having calculated $DA_{req}$, $RSU_{j}$ judges the necessity of precaching $C_{c}$ for $V_{req}$. When the total amount of $C_{c}$ is $TA_{C_{c}}$, if $TA_{C_{c}}$ ≤ $DA_{req}$, $RSU_{j}$ can download $TA_{C_{c}}$ to $V_{req}$ in its communication coverage area. Thus, $RSU_{j}$ sends data packets of $C_{c}$ to $V_{req}$ during its movement through the communication range of $RSU_{j}$. When all of the data packets have been successfully delivered to $V_{req}$, $RSU_{j}$ finishes the process of downloading $C_{c}$ for $V_{req}$.

However, if $TA_{C_{c}}$>$DA_{req}$, $RSU_{j}$ conducts the precaching of $C_{c}$ for $V_{req}$, because $RSU_{j}$ cannot fully deliver the total amount of $C_{c}$ to $V_{req}$ in its communication coverage area. For the content precaching, $RSU_{j}$ first calculates the remaining amount of $C_{c}$ after removing the downloadable amount $DA_{req}$ of $V_{req}$ in its communication coverage area. Then, the remaining amount $RMA_{C_{c}}$ is $\left(TA_{C_{c}}-DA_{req}\right)$. Having obtained $RMA_{C_{c}}$, $RSU_{j}$ needs to select a content-precaching vehicle group $L_{CPVG}$ from the vehicles in its communication coverage area for $V_{req}$ in the outage zone between $RSU_{j}$ and $RSU_{j+1}$. If the total $\sum_{n=1}^{K}PA_{n}$ of the available precaching amount $PA_{i}$ of every vehicle $V_{i}$ in the communication coverage area of $RSU_{j}$ is less than $RMA_{C_{c}}$, all vehicles are included in $L_{CPVG}$. $RMA_{C_{c}}-\sum_{n=1}^{K}PA_{n}$ becomes the RSU precaching amount $RPA_{j+1}$ of $RSU_{j+1}$. Then, $RSU_{j}$ sends a precaching packet with $RPA_{j+1}$ to $RSU_{j+1}$ in order to precache $RPA_{j+1}$, because all the vehicles in $L_{CPVG}$ cannot fully relay the total $RMA_{C_{c}}$ to $V_{req}$ in the outage zone. If $RSU_{j+1}$ successfully receives the precaching packet, it sends an ACK packet to $RSU_{j}$ and saves $RPA_{j+1}$ in its caching storage. However, if the total amount $\sum_{n=1}^{K}PA_{n}$ is larger than $RMA_{C_{c}}$, $RSU_{j}$ selects suitable vehicles in its communication coverage area to cover only $RMA_{C_{c}}$ as $L_{CPVG}$. Next, $RSU_{j}$ allocates the precaching amount for each vehicle in $L_{CPVG}$. After selecting $L_{CPVG}$ and allocating the precaching amount, $RSU_{j}$ sends a selection packet to every vehicle in $L_{CPVG}$. The selection packet for each vehicle $V_{i}$ includes $V_{i}$’s ID, $V_{req}$’s ID, and the content data with the precaching amount of $C_{c}$. On receiving the selection packet, $V_{i}$ becomes aware of its selection as a precaching vehicle and $V_{req}$ as the target for precaching. If $V_{i}$ has fully receives its precaching amount while moving in the communication coverage area of $RSU_{j}$, it sends an ACK packet to $RSU_{j}$.

After $V_{req}$ has downloaded the $DA_{req}$ of $C_{c}$ in the communication coverage area of $RSU_{j}$, when it leaves the the communication coverage area of $RSU_{j}$, it enters the outage zone between $RSU_{j}$ and $RSU_{j+1}$. Because $V_{req}$ has not fully downloaded the total amount of $C_{c}$, it downloads the remaining amount $RMA_{C_{c}}$ from the content-precaching vehicle group $L_{CPVG}$ in the outage zone. When $V_{req}$ is traveling in the outage zone, if each vehicle $V_{i}$ in $L_{CPVG}$ has downloaded its precaching amount in the communication coverage area of $RSU_{j}$ and has left this communication coverage area, $V_{i}$ checks whether it is located in the communication coverage of $V_{req}$. If $V_{i}$ has the ID of $V_{req}$ in its neighbor table due to receiving a beacon packet from $V_{req}$, it sends a relay packet with its precaching amount of $RMA_{C_{c}}$ to $V_{req}$. If $V_{req}$ has fully received the relay packet, it sends an ACK packet to $V_{i}$. By this process, $V_{req}$ receives the precaching amounts from all precaching vehicles in $L_{CPVG}$. When $V_{req}$ has fully downloaded the total amount of $RMA_{C_{c}}$ through precaching and relaying by $L_{CPVG}$, it has downloaded the total amount of $C_{c}$ and has thus finished the process of downloading $C_{c}$. However, the total $\sum_{n=1}^{K}PA_{n}$ available precaching amounts of all vehicles in the communication coverage area of $RSU_{j}$ could be less than $RMA_{C_{c}}$. Then, $V_{req}$ can download only $\left(RMA_{C_{c}}-\sum_{n=1}^{K}PA_{n}\right)$ from $L_{CPVG}$ in the outage zone. In this case, $V_{req}$ does not fully download $RMA_{C_{c}}$ from $L_{CPVG}$ in the outage zone before it reaches the communication coverage area of the next RSU $RSU_{j+1}$. When $V_{req}$ enters the communication coverage area of $RSU_{j+1}$, it sends a request packet with information about both itself (i.e., ID, location, destination, and mobility) and $C_{c}$ (i.e., ID, size, etc.) to $RSU_{j+1}$. Here, the size of $C_{c}$ is $\left(RMA_{C_{c}}-\sum_{n=1}^{K}PA_{n}\right)$ and is the same for $RPA_{j+1}$. On receiving the request packet, $RSU_{j+1}$ sends an ACK packet to $V_{req}$ in response to the request. Since $RSU_{j+1}$ has already precached $RPA_{j+1}$ from $RSU_{j}$ and has it in its caching storage, it instantly starts the content downloading process with $RPA_{j+1}$ for $V_{req}$, as in the previous RSU ($RSU_{j}$). This process is continuously repeated until $V_{req}$ has completely downloaded the total amount $TA_{C_{c}}$ of the intended content $C_{c}$. Eventually, the process of downloading $C_{c}$ for $V_{req}$ is finished.

## 5. Performance Evaluation

In this section, we evaluate the performance of the proposed scheme, MPVS, by performing a simulation on NS3. In order to evaluate the scheme’s performance in terms of the reliability and fairness in requesting a vehicle’s content, we first describe the simulation environment and matrix for comparison in Section 5.1. Then, in Section 5.2, we evaluate the performance of the proposed scheme and a comparison scheme based on the simulation results obtained in various environments. Table 2 lists the parameters used in our simulation.

### 5.1. Simulation Environment

To evaluate the performance of the proposed scheme, MPVS, we simulated requests for content in a highway scenario using NS3 [34]. On a straight road with a length of 30 km, 6 RSUs were spaced 4 km apart, and each RSU had a 1 km radius circle as its communication range. All RSUs were interconnected by backhaul links that had a transmission rate of 10 Gbps and a backhaul link latency of 10 ms. The backhaul links connected the content server to other RSUs. In addition, the RSUs had a maximum wireless transmission rate of 54 Mbps when providing requested content to vehicles based on WAVE [33], which is provided by default in NS3. To cache and precache requested and provided content, the RSUs had their own content storage, which had a size of 1 TB. Then, an average of 100 vehicles for every 1 km of road were randomly distributed on the bidirectional four-lane road at the beginning of the simulation, and they traveled on the roads of the network according to the highway mobility model, which considered a highway scenario. In this highway mobility model, the average speed of the vehicles was 80 km/h, and the acceleration of each vehicle was individually determined every second based on Gaussian distribution. Additionally, none of the vehicles stopped on the road during their travel time, and they did not exceed the legal speed limit. They could store any content up to 512 MB and request content from an RSU via WAVE when they wanted to download new content. The requests for content by each vehicle were decided every 10 s on average according to Poisson distribution. When a vehicle decided to request content based on Poisson distribution, the requested content was determined based on Zipf’s law [35] with an exponent of 0.75 and 1,000,000 considered content items. Furthermore, all vehicles had a maximum wireless transmission rate of 54 Mbps and a 200 m radius circle as their communication range when communicating with each other. This indicated the popularity of the requested content. We performed 30,000 simulations per scenario with each environmental parameter, and each simulation had a duration of 3600 s.

When selecting a comparison scheme for evaluating the proposed scheme, there were two compelling reasons to consider a scheme that incorporated similar characteristics to the proposed scheme. First, the comparison scheme needed to employ a similar mathematical model for calculating the downloadable or precaching amount of content. This ensured a fair and meaningful comparison. By selecting a scheme with a similar mathematical framework, we could directly compare the outcomes and assess the relative strengths and weaknesses of the different approaches in a consistent manner. Secondly, it was important to choose a scheme that involved multiple vehicles as precaching nodes, mirroring the scenario in which the proposed scheme operated. By selecting a comparison scheme that shared this characteristic, we could evaluate the performance of the proposed scheme under conditions that closely resembled real-world settings. Therefore, in order to evaluate the performance of the proposed scheme, we compared it with the existing V2V precaching scheme called AMRS [20]. The existing V2V precaching schemes for outage zones, including AMRS, start with the relaying vehicle that can deliver the largest amount of content and let it use all of its dwell time to precache content for the requester vehicle. The reason for this is that they do not consider the fairness for the relaying vehicles. Thus, these vehicles have no time to spend on requesting and receiving their own intended content.

To compare the proposed scheme with the AMRS scheme, we measured two metrics, as follows:

- Request success rate (%): A vehicle that is selected as a relaying vehicle cannot request or receive its own intended content because it spends its entire dwell time within the coverage area of the current RSU precaching the requested content for other requester vehicles. Therefore, the vehicle suffers a very long delay until it reaches the next RSU. To measure this request failure, we evaluated the request success rate, which is the ratio of the number of successful content requests to the total number of requests. It is directly related to user QoS and reliability because it affects delays. Thus, it is a critical factor for content delivery to vehicles in CCVNs.
- Precaching fairness (*F*): In the AMRS scheme, the relaying vehicle that has abundant dwell time spends all of its dwell time precaching, which is unfair to this vehicle. To measure the fairness for the selected relaying vehicles, we evaluated the precaching fairness based on Shannon’s diversity index as follows:
  (20) $$F=\sum_{k=1}^{K}\frac{\left(p_{dist}^{\left(k\right)}\times log\left(p_{dist}^{\left(k\right)}\right)\right)}{K},$$
  Here, $p_{dist}^{\left(k\right)}$ indicates the ratio of the precaching usage rate $p_{prec}^{\left(k\right)}$ of the *k*–th relaying vehicle to the sum of each precaching usage rate as follows:
  (21) $$p_{dist}^{\left(k\right)}=\frac{p_{prec}^{\left(k\right)}}{\sum_{k=1}^{K}p_{prec}^{\left(k\right)}},$$
  and $p_{prec}^{\left(k\right)}$ is calculated as
  (22) $$p_{prec}^{\left(k\right)}=\frac{\left(Precaching\_amount_{k}\right)}{\left(Available\_amount_{k}\right)},$$
  where $Precaching\_amount_{k}$ is the amount that the *k*–th relaying vehicle has to precache, and $Available\_amount_{k}$ is the amount of content that the *k*–th relaying vehicle can receive within the coverage area of the current RSU. Since the number of vehicles selected as relaying vehicles was the same in each scheme, a large *F* indicated a high disorder of $p_{prec}^{\left(k\right)}$ for each relaying vehicle. Furthermore, as the number of relaying vehicles increases, many vehicles have to spend their dwell time on the requests of other vehicles, causing *F* to decrease.

Then, for performance comparison, we implemented four environmental parameters, as follows:

- Request decision period (s): The request decision period is a parameter that determines how often vehicles decide to request content, and it reflects the impact on performance as the number of requests from vehicles increases. Therefore, it reflects the performance according to the number of requester vehicles when the number of candidate vehicles is fixed. A shorter decision period means that vehicles make content request decisions more frequently, leading to an increase in the number of requester vehicles that an RSU has to select as relaying vehicles. As a result, the relaying vehicles may spend more of their dwell time on the requests of other vehicles, which can affect their ability to request their own content.
- Requested content size (GB): The requested content size is the average size of the requested content and indicates the number of candidate vehicles required. Therefore, it reflects the performance according to the number of candidate vehicles required when the number of requester vehicles is fixed. If the size of the requested content is too small, the precaching fairness is less affected by selecting a single relaying vehicle because it requires fewer candidate vehicles. As the size of the requested content increases, the selection of requester vehicles differs in each scheme. If the size of the requested content is too large, each requester vehicle employs a large number of candidate vehicles as relaying vehicles, resulting in a decrease in the request success rate.
- Vehicle density (/km): The vehicle density is the number of vehicles on a 1 km stretch of the straight road. Therefore, it reflects the performance when the number of candidate vehicles and the number of requester vehicles grow at the same rate. When the number of vehicles is small, fewer requester vehicles require fewer relaying vehicles, which means that other vehicles have a higher success rate in their requests; however, this comes at the expense of fewer candidate available vehicles, so it is not possible to select a better group. If the number of vehicles is large enough that a better group can be selected, the precaching fairness of the proposed scheme is improved. However, if the number of vehicles is too large, the number of requester vehicles increases, requiring a larger number of candidate vehicles, and the request success rate decreases. For example, if the number of requesting vehicles quadruples, more candidate vehicles are required because the best groups are exhausted and the next best group must be selected from the remaining candidate vehicles.

### 5.2. Simulation Results

**Figure 2:**
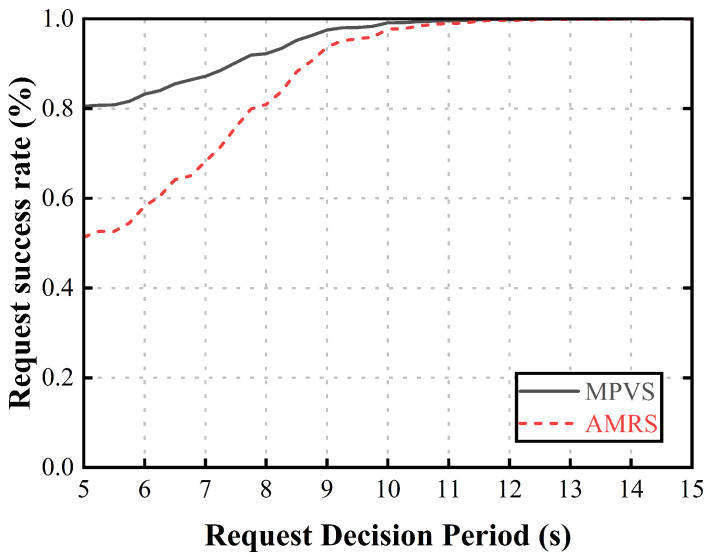
Request success rate according to the request decision period.

Figure 2 shows the request success rate according to the average period of request decisions from each vehicle. When all vehicles frequently made content requests, many vehicles were used as relaying vehicles by the increased number of requester vehicles due to the limited number of available candidate vehicles that could be included in the content-precaching vehicle group. As the request decision period increased, the number of relaying vehicles required to make up the content-precaching vehicle group was reduced by the decreasing number of requester vehicles, resulting in an improvement in the request success rate because fewer vehicles needed to be selected as relaying vehicles. In AMRS, the vehicles selected as relaying vehicles in the content-precaching vehicle group spent all of their dwell time precaching content for the requester vehicles, except for the vehicle that had the smallest available precaching amount in the group. Therefore, since almost all vehicles used as relaying vehicles could not request their own content, the request success rate of AMRS was lower than that of MPVS. In MPVS, since each selected vehicle only precached a certain amount of the requested content, they did not use all of their dwell time, making it possible for them to request their own content. This led to better performance than AMRS.

**Figure 3:**
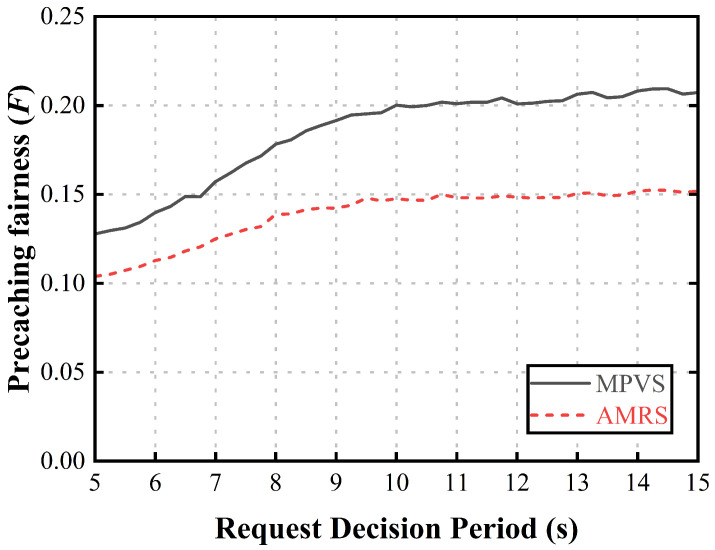
Precaching fairness according to the request decision period.

Figure 3 shows the precaching fairness according to the average period of request decisions from each vehicle. If all vehicles frequently made content requests, the required number of relaying vehicles selected from the fixed number of candidate vehicles was increased, leading to a decrease in the precaching fairness of each scheme. If the request decision period was long enough, the precaching fairness increased, because the required number of relaying vehicles was reduced. In AMRS, since almost all selected vehicles spent all of their dwell time precaching, they could not request or receive their own content. Therefore, this scheme had less precaching fairness than MPVS. In MPVS, each relaying vehicle in the content-precaching vehicle group was allocated the proper amount of requested content to precache in terms of precaching fairness. Thus, even if the number of relaying vehicles in the content-precaching vehicle group was the same in each scheme, MPVS showed improved precaching fairness compared to AMRS.

**Figure 4:**
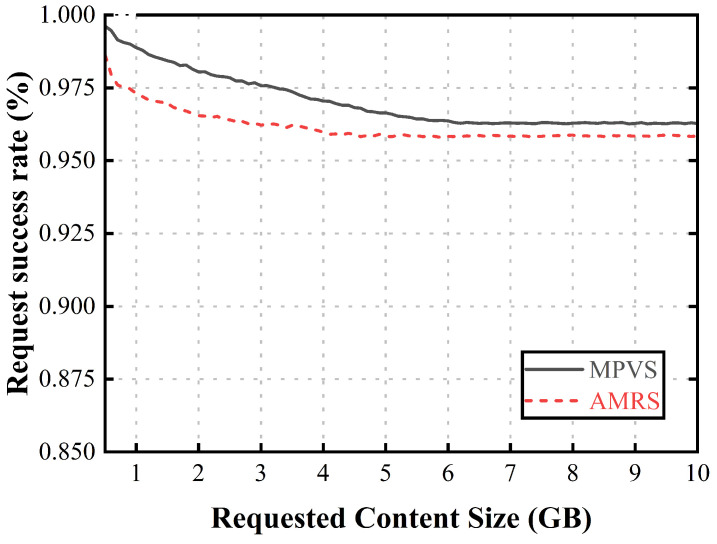
Request success rate according to the size of the requested content.

Figure 4 shows the request success rate according to the size of the requested content. When the size of the requested content was small, the current RSU could provide all the requested content to each requester vehicle. As the requested content size increased, the content-precaching vehicle group had to be selected, leading to a drop in the request success rate due to the vehicles being used as relaying vehicles. If the requested content size was too large, the request success rate did not decrease, because the remainder of the requested content was precached at the next RSU without selecting more relaying vehicles due to the limited outage zone. In AMRS, as the required number of relaying vehicles increased, more relaying vehicles spent all of their dwell time precaching the requested content for the requester vehicle. Therefore, this scheme had a low success rate. In MPVS, as the required number of relaying vehicles increased, the selected relaying vehicles had abundant dwell time to request their own content, because they were allocated a proper amount of requested content to precache in terms of precaching fairness, resulting in higher performance than AMRS.

**Figure 5:**
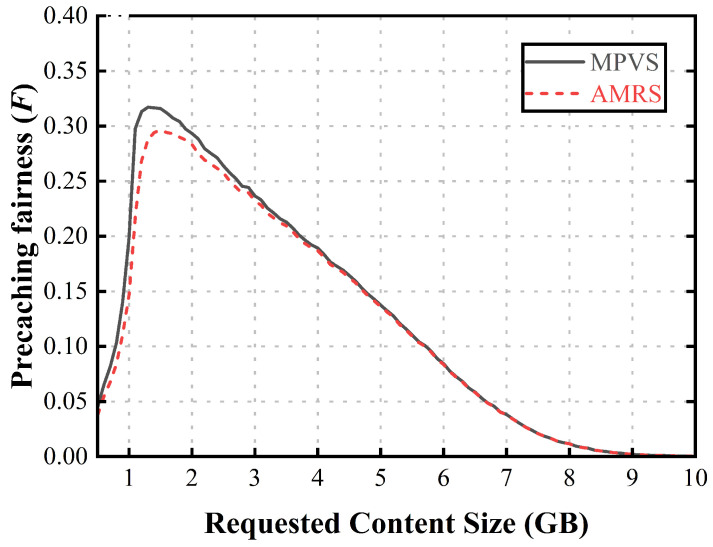
Precaching fairness according to the size of the requested content.

Figure 5 shows the precaching fairness according to the size of the requested content. When the size of the requested content was too small, only a few relaying vehicles were selected as members of the content-precaching vehicle group, because only a portion of the requested content was provided by the current RSU. If the size of the requested content was large enough, both schemes selected relaying vehicles, so there was a difference between the two schemes. However, if the requested content size was too large, the precaching fairness was almost the same, because a portion of the requested content was precached at the next RSU. Because the required number of relaying vehicles increased as the size of the requested content increased, the precaching fairness decreased. In AMRS, since all dwell time was spent on the precaching amount allocated to the selected relaying vehicles, except for the vehicle that had the smallest available precaching amount in the group, the precaching fairness was lower than that of MPVS. In MPVS, since all vehicles had abundant dwell time to request or receive their own content when the requested content size was not too large, this scheme had a higher precaching fairness than AMRS.

**Figure 6:**
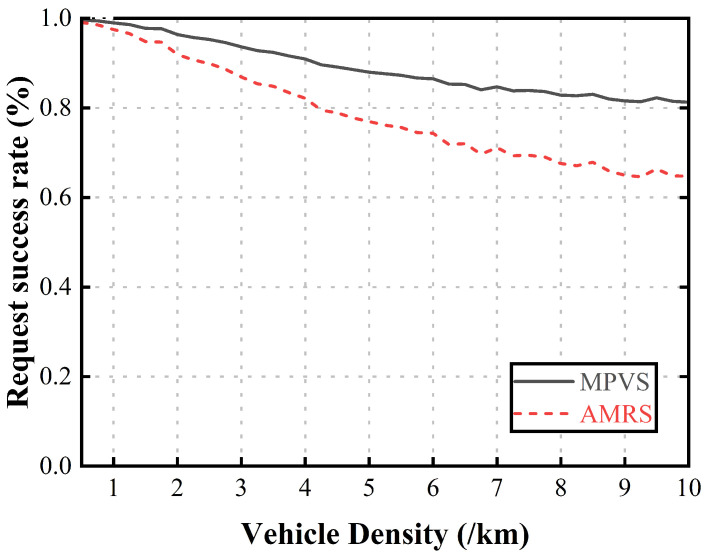
Request success rate according to the vehicle density.

Figure 6 shows the request success rate according to the vehicle density. When there were very few vehicles on the road, the number of requester vehicles was small. As few vehicles were selected as relaying vehicles, the request success rate was high. If the number of candidate vehicles was not sufficient to provide the requested content within the outage zone, the next RSU precached the remaining portion. In the case of increased vehicle density, the number of requesting vehicles was more affected than the number of candidate vehicles. Therefore, as the number of candidate vehicles selected as relaying vehicles increased, the number of vehicles that could not request or receive their own content increased. In AMRS, most of the selected relaying vehicles used their dwell time to precache content for other requester vehicles. This resulted in a drop in performance. In MPVS, if the number of candidate vehicles was insufficient to deliver the requested content within the outage zone, the selected relaying vehicles used their dwell time as in AMRS; then, the next RSU precached the remaining portion. However, as the vehicle density increased, MPVS suffered less performance degradation than AMRS due to fairness considerations.

**Figure 7:**
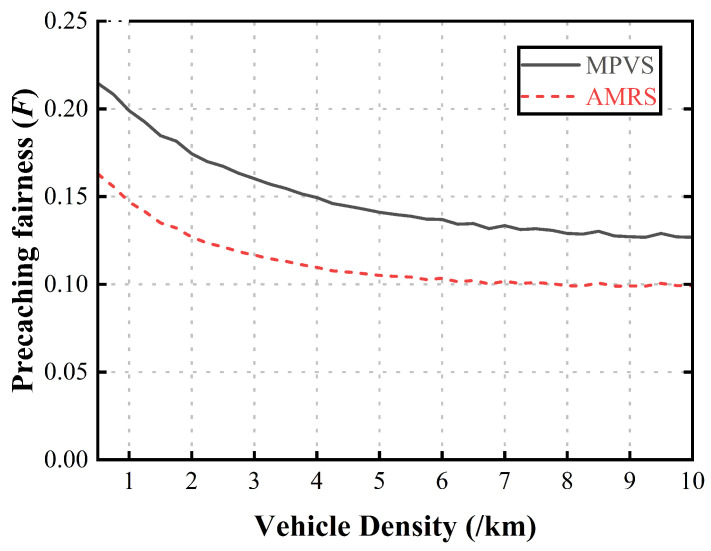
Precaching fairness according to the vehicle density.

Figure 7 shows the precaching fairness according to the vehicle density. When there were few vehicles on the road, the number of requester vehicles was small, and the schemes could select the best content-precaching vehicle group. As the vehicle density increased, the number of requester vehicles increased. Then, the later requester vehicles had to select the next best content-precaching vehicle group, because the early requester vehicles had already selected the best content-precaching vehicle group. Therefore, as the vehicle density increased, the opportunities to select the best content-precaching vehicle group decreased, leading to a decrease in the precaching fairness. In AMRS, the selected relaying vehicles used their dwell time to precache content for other requester vehicles, resulting in less precaching fairness than in MPVS. MPVS achieved better performance than AMRS because the selected relaying vehicles were assigned to precache an appropriate portion of the requested content by considering the fairness. In addition, since more than one relaying vehicle was selected because the content size was large enough, the performance difference was notable.

## 6. Conclusions

In conclusion, the high-speed nature of ICVNs presents challenges in meeting increased content demands and addressing outage zones where RSU coverage may be limited. Existing V2V precaching schemes have focused on delivering the maximum amount of content to the requester vehicle within the outage zone, but this approach often results in relaying vehicles consuming all their dwell time, leaving no opportunity for them to request or receive their own content. To address this issue, we proposed the MPVS scheme, which ranks a group of candidate vehicles that can serve as relaying vehicles and allocates them to precache a fair amount of content. First, we modeled the amount of content that could be downloaded and precached by the candidate vehicles with mathematical formulas and used these formulas to select the content-precaching vehicle group that could provide the maximum amount of content to the requester vehicle. Then, we ensured fairness by allocating an equal proportion of the requested content to each relaying vehicle in the selected group. To evaluate the performance of our proposed scheme, we designed a highway mobility model with a straight road, wherein vehicle speed and acceleration followed a Gaussian distribution. We also designed a content request model based on Poisson distribution for content request decision periods, Zipf’s law for content popularity, and Gaussian distribution for content size. The simulation results in various environments showed that our proposed MPVS scheme achieved greater fairness compared to the existing AMRS V2V precaching scheme, indicating an improved request success rate.

However, there are several areas that could be explored further to enhance the feasibility and applicability of our research. First, we could consider a realistic mobility model in an urban scenario. Therefore, as a next step, we intend to extend our research to urban scenarios, where vehicular networks face distinct challenges due to high-density traffic; complex road networks (featuring traffic lights, vehicle direction changes, etc.); and heterogeneous communication environments. By considering the unique characteristics of urban environments, such as traffic congestion and signal interference, we could develop tailored solutions to optimize content delivery, mitigate latency, and improve overall network performance for real scenarios. Additionally, we could consider a realistic content consumption model. To further enhance the realism of our research, it is essential to develop a more accurate content consumption model. In practice, content popularity, user preferences, and content caching behavior can be dynamic and complex. Furthermore, users often decide after a short time to stop watching the requested content (e.g., because they do not like it) and proceed to request other content. By incorporating real-world data and considering factors such as user behavior, content dynamics, and temporal variations, we could create more realistic content consumption models, leading to improved performance evaluations and more accurate predictions. In addition, we could use machine learning to solve real-world problems and improve various performance aspects.

## Figures and Tables

**Table 1:** Notation for MPVS.

Notation	Description
$RSU_{j}$	The *j*-th RSU, j∈{1,⋯,J}
$V_{i}$	The *i*-th vehicle, i∈{1,⋯,I}
$C_{c}$	The *c*-th content, c∈{1,⋯,C}
$V_{req}$	The requester vehicle
$v_{i}$	The speed of $V_{i}$
$R_{I2V}$	The transmission rate of I2V
$r_{c}$	The communication range of the vehicles
$RT_{req}$	The remaining time expected for the requester vehicle to be traveling
	within the coverage area of the current RSU
$DA_{req}$	The amount that $V_{req}$ can download from the current RSU
$TA_{C_{c}}$	The total content amount of $C_{c}$
$RMA_{C_{c}}$	The remaining amount of $C_{c}$
$DT_{i}$	The downloading time of $V_{i}$ within the coverage area of the current RSU
$t_{max}$	The maximum time spent by the requester vehicle within the outage zone
$PDA_{i}$	The amount that can be downloaded by $V_{i}$ within the coverage area of the current RSU
$RA_{i}$	The amount that can be relayed by $V_{i}$ within the coverage area of the current RSU
$PA_{i}$	The amount that can be precached by $V_{i}$
$L_{CPVG}$	The content-precaching vehicle group
$TPA_{L_{CPVG}}$	The total available precaching amounts of all vehicles in $L_{CPVG}$
$RPA_{j}$	The amount precached by $RSU_{j}$
$SR_{j}$	The sets wherein every element has a sufficient $PA_{i}$
$LR_{j}$	The sets with the minimum number of elements among $SR_{j}$

**Table 2:** Simulation parameters.

Parameter	Value
Simulation time	3600 s
Network size	30 km
Vehicle mobility model	Highway mobility model
Backhaul link latency	10 ms
Backhaul link rate	10 Gbps
Maximum transmission rate of an RSU	54 Mbps
Maximum transmission rate of a vehicle	54 Mbps
An exponent of content popularity	0.75
RSU transmission range	1 km
Vehicle transmission range	200 m
Vehicle’s CS	512 GB
RSU’s CS	1 TB
Distance between RSUs	4 km
Vehicles’ average speed	80 km/h
Mean of request decision frequency	[5, 15] s
Size of requested content	[0.5, 10] GB
Vehicle density	[0.5, 10] per km

## Data Availability

Not applicable.
